# Effects of bacterial translocation on hemodynamic and coagulation parameters during living-donor liver transplant

**DOI:** 10.1186/s12871-018-0507-7

**Published:** 2018-04-25

**Authors:** Heba A. Moharem, Fawzia Aboul Fetouh, Hamed M. Darwish, Doaa Ghaith, Mohamed Elayashy, Amr Hussein, Riham Elsayed, Mohammad M. Khalil, Amr Abdelaal, Mahmoud ElMeteini, Ahmed Mukhtar

**Affiliations:** 1 Department of Anesthesia, Surgical Intensive Care and Pain Management, Faculty of Medicine, Alexandria University, Alexandria, Egypt; 2 Department of Anesthesia, Surgical Intensive Care and Pain Management, Faculty of Medicine, Cairo University, 1 Alsaray st, Almanial, Cairo, Egypt; 3 Department of clinical and chemical pathology, Faculty of Medicine, Cairo University, Cairo, Egypt; 4 Department of surgery, Ainshams University, Cairo, Egypt

**Keywords:** Bacterial translocation, Liver transplantation, Bacterial DNA, Coagulation factors

## Abstract

**Background:**

Bacterial translocation (BT) has been proposed as a trigger for stimulation of the immune system with consequent hemodynamic alteration in patients with liver cirrhosis. However, no information is available regarding its hemodynamic and coagulation consequences during liver transplantation.

**Methods:**

We screened 30 consecutive adult patients undergoing living-donor liver transplant for the presence of BT. Bacterial DNA, Anti factor Xa (aFXa), thromboelastometry, tumor necrosis factor-α TNF-α, and interleukin-17 (IL-17) values were measured in sera before induction of anesthesia. Systemic hemodynamic data were recorded throughout the procedures.

**Results:**

Bacterial DNA was detected in 10 patients (33%) (bactDNA(+)). Demographic, clinical, and hemodynamic data were similar in patients with presence or absence of bacterial DNA. BactDNA(+) patients showed significantly higher circulating values of TNF-α and IL-17, and had significantly higher clotting times and clot formation times as well as significantly lower alpha angle and maximal clot firmness than bactDNA(−) patients, *P* < 0.05. We found no statistically significant difference in aFXa between the groups, *P* = 0.4. Additionally, 4 patients in each group needed vasopressor agents, *P* = 0.2. And, the amount of transfused blood and blood products used were similar between both groups.

**Conclusion:**

Bacterial translocation was found in one-third of patients at the time of transplantation and was largely associated with increased markers of inflammation along with decreased activity of coagulation factors.

**Trial registration:**

Trial Registration Number: NCT03230214. (Retrospective registered). Initial registration date was 20/7/2017.

## Background

Bacterial translocation (BT) is defined as translocation of bacteria and/or bacterial products from the gut to the mesenteric lymph nodes [1]. Although BT is a physiologically controlled process in healthy subjects, it is considered pathological in patients with liver cirrhosis who sustain increased BT events [2]. The clinical significance of diagnosing BT in patients with liver cirrhosis has been addressed [1–4]. Most studies have found that the presence of BT in cirrhotic patients is associated with significant hemodynamic changes, even in the absence of clinical infection, and is due to the release of inflammatory mediators like tumor necrosis factor-α (TNF-α) [2, 3].

The effects of BT on coagulation abnormalities in patients with liver cirrhosis have not been investigated. Studies examining the relationship between true bacterial infection and coagulopathy have found that the presence of infection increases the incidence of bleeding in patients with liver cirrhosis [5, 6]. The mechanism of this infection-induced coagulopathy remains poorly understood, but one postulated mechanism is that bacterial infection creates heparinoid-like substances [6]. These endogenous anticoagulants have been confirmed by thromboelastography and by the presence of anti-factor X activity in the blood of infected patients [5, 6].

The aim of the present study was primarily to explore the incidence of BT in cirrhotic patients at the time of liver transplantation, and secondarily to investigate the effect of BT on hemodynamic, inflammatory, and coagulatory parameters during living-donor liver transplantation.

## Methods

Thirty consecutive adult patients with grade C liver cirrhosis undergoing living-donor liver transplant were enrolled in the study. The Research Ethics Committee approved the study protocol and written informed consents were obtained from all participating patients. Patients under 18 years, those who had positive blood or ascitic fluid cultures or who underwent treatment with antibiotics in the preceding 2 weeks, and those with fulminant liver failure were all excluded from the study.

A standardized anesthetic protocol was used [7]. Anesthesia was induced with intravenous propofol, fentanyl, and atracurium. Anesthesia was maintained with sevoflurane adjusted between 1 and 2% in an oxygen/air mixture, a fentanyl infusion at 1–2 μg/kg/hr, and an atracurium infusion at 0.5 mg/kg/hr. Mechanical ventilation was provided by a Primus anesthesia machine (Dräger, Germany) using a tidal volume of 8 mL/kg with the respiratory rate adjusted to maintain PaCO2 between 30 and 35 mmHg. All patients were monitored for temperature, noninvasive and invasive arterial blood pressure, 5-lead electrocardiogram, peripheral oxygen saturation, end-tidal carbon dioxide tension, hourly urinary output, central venous pressure (CVP), and pulmonary artery occlusion pressure (PAOP). A pulmonary artery catheter (PAC) (OPTIQ SVO2/CCO Abbott Laboratories, North Chicago, IL, USA) was inserted into the right internal jugular vein. All the patients received 6 ml/kg crystalloids as maintenance intraoperative fluid. Fluid resuscitation was guided by using the pulse pressure variations (PPVs) through a Philips Intellivue MP 70 monitor (Philips, Suresnes, France). PPV more than 13% indicated that patients were fluid-responsive, and cardiac output could be increased by additional intravenous fluid administration. The patients received 250 ml boluses of 5% albumin as needed to maintain a PPV < 13%. Blood transfusions were administered based on the hemoglobin level (< 7 g/dl), and a thromboelastometry was used to choose blood transfusion products (platelets, fresh frozen plasma (FFP), and cryoprecipitate). Transfusion of FFPs was required when EXTEM clotting time (CT) is > 80 s. Transfusion of cryoprecipitate is indicated if EXTEM maximal clot firmness (MCF) < 35 mm and FIBTEM MCF < 8 mm. If EXTEM MCF < 35 mm and FIBTEM MCF > 8 mm this indicates the need for platelet transfusion [8]. In all cases, the decision of transfusion depends on the results of thromboelastometry and the presence of clinically significantly bleeding. We typically transfused FFPs at dose of 10–15 ml/kg but in 2 unit-increments till the bleeding cease. Norepinephrine was administered if the mean arterial pressure was < 70 mmHg despite adequate volume resuscitation.

### Measurements

#### Hemodynamic variables

Heart rate, mean arterial blood pressure, PAOP, CVP, and cardiac output (using a pulmonary artery catheter) were monitored. Hemodynamic data were recorded after induction of anesthesia, at the end of the preanhepatic phase, at the end of the anhepatic phase, and at the end of the surgery.

#### Laboratory data

Whole blood samples were taken from the patients before induction of anesthesia to perform the necessary tests.

#### Thromboelastometry

EXTEM, INTEM, and HEPTEM tests were performed with ROTEM delta (ROTEM®). The following four variables were recorded for each test: CT, clot formation time (CFT), alpha angle (α angle), and MCF. For the FIBTEM test, only the MCF was documented.

#### Cytokine levels

Serum levels of IL-17A and TNF-α were determined using the enzyme-linked immunosorbent assay (ELISA) kits of Euroclone (Wetherby, Yorkshire, UK) for IL-6 and TNF, and the R&D Systems kit (Wiesbaden, Germany) for IL-17, according to the manufacturer’s instructions.

#### Activated factor X (aFXa)

The level of aFXa activity was determined using a validated chromogenic assay kit (COAMATIC Heparin; Chromogenix, Instrumentation Laboratory Company, Lexington, KE, USA) with the substrate S-2732 and the recommended apparatus (STA-R Evolution; Diagnostica Stago, Asnières, France). The test was considered positive when the level of anti-Xa was > 0.2 units/ml.

#### Bacterial blood culture and DNA extraction

We incubated 5–10 ml (optimally 8–10 ml) blood in a BACTEC 9120 system (Becton-Dickinson). All blood culture bottles (BACTEC™ Plus Aerobic/F and BACTEC™ Plus Anaerobic/F Becton-Dickinson) containing resins were incubated for a minimum of 5 days according to the manufacturer’s instructions. When a positive signal was detected, bottles were removed and an aliquot of the broth was Gram-stained and processed by a range of routine biochemical test methods*.* Bacterial DNA was extracted from blood culture samples using the QIAmp DNA Minikit (Qiagen) according to the protocols in the manufacturer’s instructions. The extracted DNA was stored at 4 °C until required for PCR. We used the Dream Taq TM PCR Master Mix 2X (Fermentas) (#K1071) containing: Dream Taq TM DNA Polymerase, Dream Taq TM PCR Buffer, 4 mM MgCl_2_, and dNTPs for the PCRs.

Each reaction tube contained: Master Mix 12.5 μl, 0.2 mM of each primer (amount of Primer Mix 2 μl; 1 μl forward & 1 μl reverse each diluted 1:10 from the stock), template DNA 10 μl (approximately 500 ng), and 5 μl 1X PCR buffer. The reaction mixtures were vortexed briefly. Amplification reactions were carried out in a Seegene (SEE AMP) thermocycler.

### Other data collection

We also kept records of Child-Pough (CTP) scores, Model for End Stage Liver Disease (MELD) scores, graft weight ratios (GWRs), and the use of intravascular volume replacement therapy [including colloid infusion and transfusions of packed red blood cells (PRBCs) and FFP]. All complications including rejection episodes, graft dysfunction, renal replacement therapy, nosocomial infections, hospitalization length, and ICU length of stay were documented.

### Statistical analysis

Sample size estimation was based on the presence of anti-Xa activity because it is the main outcome variable. Previous study found that anti-Xa was present in 6.7 and 60% of non-infected and infected cirrhotic patient respectively [5]. Considering the incidence of bacterial translocation is 30%. We estimated the sample size to be 30 patients with power of 0.8 and alpha error 0.05 [2].

Descriptive statistics of the baseline characteristics, ROTEM, cytokines and anti-Xa values are expressed as median (interquartile range (IQR)). The Mann–Whitney rank-sum test (two-tail) was used for comparison of continuous variables between bacterial DNA(+) and bacterial DNA(−) cases. For categorical data, Fisher exact or chi-square tests were used for comparison as appropriate. A *P* value ≤0.05 was considered statistically significant.

## Results

Thirty patients were enrolled in the study. Bacterial DNA (bactDNA) was only detected in 10 patients (33%). Patients were divided into two groups according to the presence or absence of bacterial DNA. There were no significant differences between the two studied groups in terms of age, gender, body mass index (BMI), MELD or CTP scores. Also, we found no significant differences in terms of the GWR, ICU length of stay, hospitalization length, or the mortality rates (Table 1). Four patients (40%) in the bactDNA(+) group and 6 (30%) in the bactDNA(−) group developed nosocomial infections after the liver transplant (*P* = 0.6).

**Table 1:** Patients’ characteristics and perioperative data. Data are presented as median (IQR), ratio, or number (%)

Variables	bactDNA(−) (*n* = 20)	bactDNA(+) (*n* = 10)	*P* value
Age	51 (48–56)	58 (46–62)	0.2
Gender F/M	2/18	2/8	1.0
BMI			
MELD	17 (15–19)	18 (16–25)	0.2
Child-Pough B/C	3/17	2/8	1.0
GWR	1.2 (1.1–1.3)	1.1 (0.9–1.2)	0.15
ICU stay	8 (7–13)	8 (6–17)	0.9
Hospital stay	16 (11–21)	18 (16–22)	0.5
Mortality	3 (15%)	2 (20%)	1.0

*F/M* female/male, *BMI* body mass index, *MELD* Model for end stage liver disease, *GWR* graft weight ratio, *ICU* intensive care unit

**Fig. 1:**
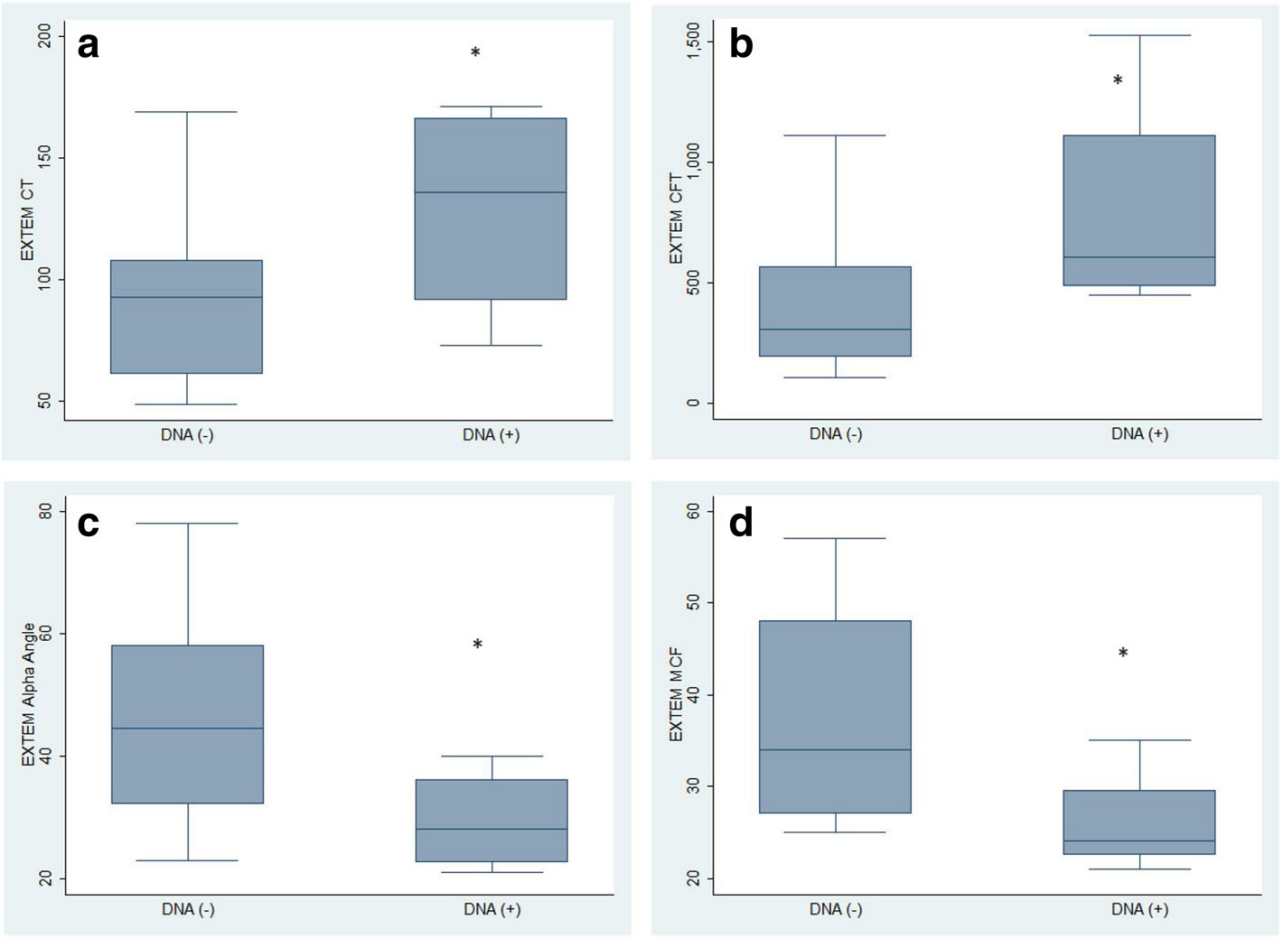
EXTEM test. **a** clotting time (CT), **b** clot formation time (CFT), **c** alpha angle, **d** maximum clot firmness (MCF). * denotes significance relative to other group

**Fig. 2:**
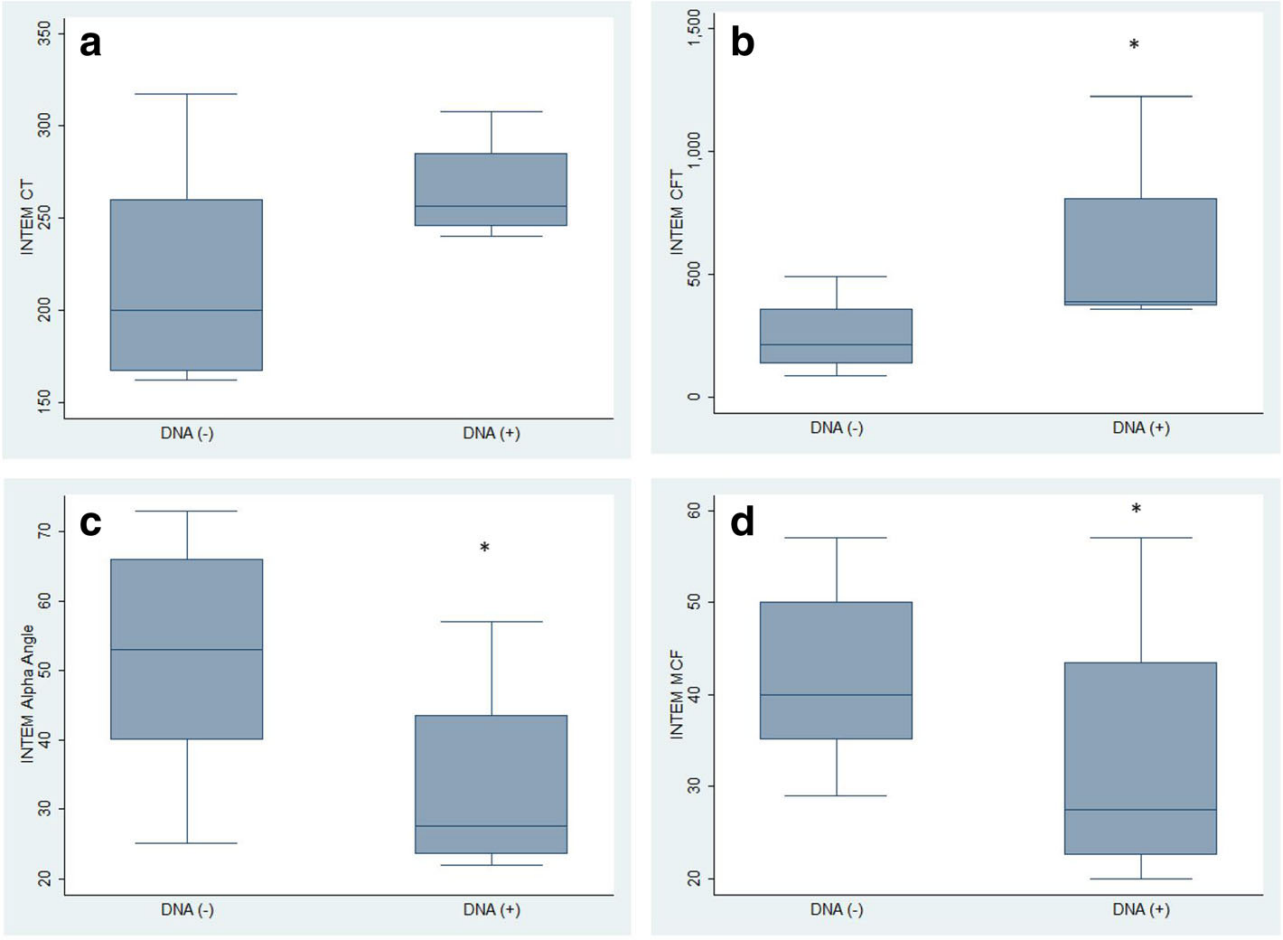
INTEM test. **a** clotting time (CT), **b** clot formation time (CFT), **c** alpha angle, **d** maximum clot firmness (MCF). * denotes significance relative to other group

EXTEM tests in bactDNA(+) patients showed a significantly prolonged CT and CFT, as well as decreased alpha angles and MCFs than bactDNA(−) patients. Similar results could be observed with the INTEM test, where CFT was prolonged and MCF was significantly reduced in bactDNA(+) patients (Figs. 1 and 2). No differences in the clotting time were noted among the HEPTEM or the INTEM assay results. Furthermore, the FIBTEM tests showed a significantly decreased MCF in bactDNA(+) patients compared with that in bactDNA(−) patients [Median (IQR) [20 (15–28) and 30 (26–38), respectively, *P* = 0.015].

### Laboratory, hemodynamic, and transfusion parameters

BactDNA(+) patients showed significantly higher circulating values of TNF-α and IL-17. Six bactDNA(−) patients (30%) and 5 bactDNA(+) patients (50%) were positive with the aFXa assay (*P* = 0.4) (Table 2), but we did not find any significant difference in hemodynamics between both groups (Table 3).

**Table 2:** Proinflammatory markers and anti-factor X. Data are presented as median (IQR) or number (percentage)

Variables	DNA (−) (*n* = 20)	DNA (+) (*n* = 10)	*P* value
TNF-α	20 (11–33)	29 (13–96) ^*^	0.002
IL-17	52 (43–52)	72 (40–118) ^*^	0.02
aFXa activity	6 (30%)	5 (50%)	0.4

*TNF-α* Tumor necrosis factor alpha, *IL-17* interleukin-17

^*^denotes significance. *P* < 0.05

**Table 3:** Hemodynamic data. Values are reported as median (IQR)

	Baseline	2 h after hepatic artery clamp	End of anhepatic phase	30 min after declamping
HR (beats/min)				
bactDNA(−)	81(71–90)	90(79–102)	86(74–105)	92(76–100)
bactDNA(+)	82(71–93)	87(74–99)	86(70–99)	82(71–93)
MAP (mmHg)				
bactDNA(−)	86(78–108)	75(70–85)	80(72–87)	77(70–91)
bactDNA(+)	82(77–95)	80(77–86)	73(69–76)	86(76–92)
CO (L/min)				
bactDNA(−)	8(7.6–9.4)	8.5(7–10)	8.25(6–9)	7.2(5.7–9)
bactDNA(+)	8(7–9)	8(7–10)	7(6.7–9.5)	8(6–9)

*HR* heart rate, *MAP* mean arterial pressure, *CVP* central venous pressure, *PAOP* pulmonary artery occlusion pressure, *CO* cardiac output

The use of vasopressors, PRBCs, and FFPs did not differ between the two groups either (Table 4).

**Table 4:** Transfusion and use of vasopressors. Data are presented as median (IQR) or number (percentage)

Variables	DNA (−) (*n* = 20)	DNA (+) (*n* = 10)	*P* value
PRBCs	3 (2–4)	2.5 (2–4)	0.2
FFPs	2 (2–3)	3 (2–6)	0.06
Use of vasopressors (%)	4 (20%)	4 (40%)	0.2

*PRBCs* Packed red blood cells, *FFPs* fresh frozen plasma

## Discussion

The main finding of this study was that cirrhotic bactDNA(+) patients who underwent liver transplant showed marked hypocoagulability on the thromboelastometric analysis, without evidence for increased endogenous heparin-like substance activity. Moreover, the presence of bacterial DNA was associated with a more systemic inflammatory response as suggested by the greater increases in TNF-α and IL-17.

One-third of our patients had bacterial translocations, as evidenced by the presence of bacterial DNA in their serum at the time of liver transplant. The incidence of bacterial translocations among cirrhotic patients had been addressed previously and was found to be 38% [2]. To the best of our knowledge, this is the first study to investigate the incidence of bacterial translocations among liver transplant recipients.

According to our findings, the bactDNA(+) patients exhibited a significant increase in proinflammatory mediators, as represented by increased levels of IL-17 and TNF-α. Consistent with this, studies have shown increased levels of inflammatory cytokines in cirrhotic patients with bacterial translocations [2, 9]. The association between high IL-17 levels and the presence of bacterial translocation remains unclear, but an increased intestinal bacterial colonization can stimulate the Paneth cells to secrete IL-17 [10]. IL-17 has been linked to the severity of inflammation in tissues by its induction of the production of other proinflammatory mediators such as IL-1, TNF, IL-6, IL-8, CCL20, and G-CSF, collectively resulting in an influx of neutrophils [11].

With ROTEM, defects of extrinsic or intrinsic pathways may be evaluated through EXTEM and INTEM, respectively. Generally, a prolongation of CT is due to a coagulation initiation defect. An isolated prolongation of CT in INTEM may subtend an intrinsic pathway defect (factors XII, XI, IX, VIII), while an isolated prolongation of CT in EXTEM may subtend an extrinsic pathway defect (factor VII plus tissue factor). On the other hand, prolongation of CFT and reduction of MCF is mainly due to a substrate deficit (e.g. fibrinogen and platelets) [12]. In the present study bactDNA(+) patients had a significant hypocoagulable state, as suggested by prolonged of CT in EXTEM and of CFT in INTEM and EXTEM and reduction of MCF amplitude in INTEM, EXTEM, and FIBTEM.

No previous studies have examined the effect of bacterial translocation on the coagulation state of cirrhotic patients. Circulating endotoxins are seems to be important predisposing factor for clotting because of endothelial dysfunction and nitric oxide dysregulation. On the other hand, several studies have shown increases in the incidence of coagulopathy in cirrhotic patients with active bacterial infections due to the presence of heparin-like substances [5, 6]. That’s why it is possible to have both bleeding and thrombosis in sequential fashion in a short time frame [13]. Anti-Xa concentrations can be measured to detect heparin activity in infected cirrhotics [5]. In our study, anti-Xa activity was comparable among patients of both groups; moreover, no differences in the clotting time were noted among the HEPTEM and the INTEM tests. This suggests that the hypocoagulable state in this group of patients cannot be explained by the presence of heparin-like substances. Plausible explanations include a sustained exposure of bactDNA(+) patients to exaggerated inflammatory responses leading to inappropriate activation and spending of coagulation factors. A similar finding is seen in patients with sepsis in whom activation of coagulation is associated with an initial hypercoagulation state that can develop into hypocoagulation as the coagulation factors become depleted [14].

In this study, the average numbers of transfused PRBCs were similar between groups; however, we evidenced a trend for high FFPs transfusions among bactDNA(+) patients.

Improvements in the anesthetic and surgical practices have led to an increasing number of patients being able to undergo LTs without the need for transfusion of red blood cells or blood products [15]. The use of a cell saver, restrictive fluid strategy, and lower limit of transfusion triggers, and the use of splanchnic vasoconstrictors have contributed effectively to the minimization of transfusions during liver transplants [7, 16]. This is the reason why the presence of other factors that impair coagulation does not appear to contribute significantly to the bleeding risk [17].

Another study found that cirrhotic bactDNA(+) patients had a lower mean arterial pressure and lower systemic vascular resistance than bactDNA(−) patients [2]. And, the difference in hemodynamic profiles should be related to increased nitric oxide levels [18]. However, in the present study we could not find any significant difference between patients with and without bacterial translocation, although we saw a trend toward higher use of vasopressors in the bactDNA(+) patients.

The postoperative course, nosocomial infection rate, and incidence of mortality were comparable between both groups of patients. However, we are aware that our study population was not big enough to detect all the significant differences between the two groups.

Given the observational nature of our study, we could not infer a cause-effect relationship between the presence of bacterial DNA and the changes in thromboelastometric parameters. Also, because of the small sample size we cannot draw any conclusions regarding the effect of bacterial translocation on either transfusion requirement or its effect on the development of postoperative organ dysfunction.

## Conclusion

Our data suggest that bacterial translocation occurs in one-third of patients at the time of transplantation and is associated with increases in inflammation markers, along with a decreased activity of coagulation factors. Further larger studies are warranted to explore the relevance of these findings with regards to the transfusion requirements and postoperative outcomes.

## Abbreviations

aFXa: Anti factor X
bactDNA: Bacterial DNA
BMI: Body mass index
BT: Bacterial translocation
CFT: Clot formation time
CO: Cardiac output
CT: Clotting time
CTP: Child-Pough scores
CVP: Central venous pressure
DNA: Deoxyribonucleic acid
ELISA: Enzyme-linked immunosorbent assay
FFP: Fresh frozen plasma
G-CSF: Granulocyte colony stimulating factor
GWR: Graft weight ratio
HR: Heart rate
IL: Interleukin
MAP: Mean arterial pressure
MCF: Maximal Clot Firmness
MELD: Model for End Stage Liver Disease
PAC: Pulmonary artery catheter
PAOP: Pulmonary artery occlusion pressure
PCR: Polymerase chain reaction
PPV: Pulse pressure variations
PRBCs: Packed red blood cells
ROTEM: Rotating thromboelastometry
TNF-α: Tumor necrosis factor, alpha
α angle: Alpha angle
